# Reperfusion Promotes Mitochondrial Biogenesis following Focal Cerebral Ischemia in Rats

**DOI:** 10.1371/journal.pone.0092443

**Published:** 2014-03-25

**Authors:** Yuying Xie, Jun Li, Guibo Fan, Sihua Qi, Bing Li

**Affiliations:** 1 Department of Anesthesiology, The Fourth Affiliated Hospital, Harbin Medical University, Harbin, China; 2 Department of Nephrology, The Second Affiliated Hospital, Harbin Medical University, Harbin, China; School of Pharmacy, Texas Tech University HSC, United States of America

## Abstract

**Background and Purpose:**

Reperfusion after transient cerebral ischemia causes severe damage to mitochondria; however, little is known regarding the continuous change in mitochondrial biogenesis during reperfusion. Mitochondrial biogenesis causes an increase in the individual mitochondrial mass of neurons and maintains their aerobic set-point in the face of declining function. The aim of this study was to examine mitochondrial biogenesis in the cortex during reperfusion following focal cerebral ischemia.

**Methods:**

Male Wistar rats were subjected to transient focal cerebral ischemia. The relative amount of cortical mitochondrial DNA was analyzed using quantitative real-time PCR at 0 h, 24 h, 72 h, and 7 d after reperfusion. Three critical transcriptional regulators of mitochondrial biogenesis were measured by semi-quantitative reverse-transcription PCR. The protein expression of cytochrome C oxidase subunits I and IV was detected by Western blotting.

**Results:**

Evidence of increased mitochondrial biogenesis was observed after reperfusion. The cortical mitochondrial DNA content increased after 24 h, peaked after 72 h, and maintained a high level for 7 d. The cortical expression of three critical genes for the transcriptional regulation of mitochondrial biogenesis, namely, peroxisome proliferator-activated receptor coactivator-1α, nuclear respiratory factor-1, and mitochondrial transcription factor A, also increased at 24 h and 72 h. The expression of peroxisome proliferator-activated receptor coactivator-1α returned to the baseline level at 7 d, but two other factors maintained higher levels compared with the controls. Moreover, the expression of cytochrome C oxidase subunits I and IV was increased in the cortex.

**Conclusions:**

These results indicate that reperfusion increased mitochondrial biogenesis following focal cerebral ischemia, and this tendency was exacerbated as the reperfusion time was extended. Reperfusion-induced mitochondrial biogenesis was mediated through up-regulation of critical transcriptional regulators of mitochondrial biogenesis.

## Introduction

Essential roles of mitochondria include regulating energy metabolism, generating reactive oxygen species (ROS), and mediating apoptosis in response to several cerebral conditions such as cerebral ischemia, brain trauma, and chronic neurodegenerative diseases [1], [2], [3], [4], [5]. The mitochondrial mass increases and the aerobic set-point is maintained when neurons decline in function [5]. Several lines of evidence have shown that mitochondria are damaged during ischemic brain injury [6], [7]. Further evidence has revealed that mitochondrial biogenesis is stimulated by ischemic injury [8]. Reperfusion is the recirculation of blood flow following transient ischemia and may be essential for the survival of ischemic brain tissue. However, reperfusion contributes to substantially more damage compared with permanent occlusion [9]. Reperfusion enhances the production of ROS, disrupts calcium homeostasis, and induces inflammatory responses, which have profound effects on cellular bioenergetics in reversible stroke [10]. Mitochondria are affected by the cascade of events following cerebral ischemic reperfusion (I-R). Evidence has shown that mitochondrial dysfunction aggravates neuronal injury after I-R because nerve cells are greatly dependent on mitochondria to support their high energy demand [11]. In a previous study, we showed that mitochondrial dysfunction occurs during the reperfusion period following 2 h of focal cerebral ischemia in rats [12]. However, the mechanism by which mitochondrial biogenesis is altered during the reperfusion period following 2 h of focal cerebral ischemia remains unclear.

The abundance of mitochondria is determined by biogenesis and the division of organelles [13], and the coordination of several mechanisms is required during the process of mitochondrial biogenesis. Basic mechanisms include the expression of mitochondrial and nuclear genes, mitochondrial protein expression and import, the regulation of mitochondrial fission and fusion, and mitochondrial turnover in response to various stimuli [14]. Several transcriptional regulators are involved in the process of mitochondrial biogenesis, three of which play an important role in regulating mitochondrial biogenesis. Peroxisome proliferator-activated receptor γ coactivator-1α (PGC-1α) integrates physiological signals to enhance mitochondrial biogenesis [15] and is a master regulator of ROS-scavenging enzymes [16]. Nuclear respiratory factor 1 (NRF-1), which was the first isolated mammalian transcription factor common to the expression of nuclear respiratory genes, functions as a positive regulator of transcription [17], [18]. Mitochondrial transcriptional factor A (TFAM) binds to mitochondrial deoxyribonucleic acid (mtDNA) and stimulates its transcription [19].

Numerous studies have demonstrated that ischemic or hypoxic injury increases mitochondrial biogenesis [20], [21]. However, no studies have observed mitochondrial biogenesis continuously during reperfusion. To address this issue, in the current study, mitochondrial biogenesis was observed during the reperfusion period following 2 h of middle cerebral artery occlusion (MCAO). We analyzed the mitochondrial number and the mtDNA content at various time points after reperfusion following 2 h of MCAO. We also examined the expression of three mitochondrial biogenesis factors and two related proteins during the reperfusion period.

## Materials and Methods

### Animal Preparation and Experimental Groups

All animal protocols were approved by the Committee on the Guidelines for Animal Experiments of Harbin Medical University, and all rats were handled according to the National Institutes of Health Guidelines for the Care and Use of Laboratory Animals. Adult male Wistar rats that weighed 250–280 g were used (8–9 weeks old). The rats were divided randomly into five groups (total n = 168), including: the I-R 0 h, I-R 24 h, I-R 72 h, and I-R 7 d groups. The I-R 0 h group underwent within 2 h of MCAO (n = 34). The other I-R groups underwent 2 h of MCAO followed by 24 h, 72 h, or 7 d of reperfusion (n = 34 in each group). In addition, a sham-operated group (n = 32) was used as the control (Table S1).

### MCAO Model

MCAO was performed as previously described with minor modifications [12], [22]. Briefly, all rats were fasted for 8 to 12 h with water provided *ad libitum*. The rats were anesthetized using intraperitoneal injections of chloral hydrate (dissolved in distilled water) at a dose of 350 mg/kg. A caudal arterial catheter was inserted, and the blood pressure of the animals was monitored continuously. The left common carotid artery, internal carotid artery, and external carotid artery were subsequently exposed via a midline incision in the neck. A 4-0 monofilament Nylon thread (40–3734 PK 10, Doccol Corporation Pennsylvania Ave., Redlands, CA, USA) with a silicon-rubber-coated tip was inserted into the internal carotid artery approximately 20 to 22 mm from the bifurcation. The thread entered via the external carotid artery stump and was gently advanced to occlude the middle cerebral artery. After 2 h of MCAO, the suture was removed to restore the blood flow (reperfusion was confirmed by laser Doppler). Adequate precautions were taken to prevent infection. During the surgical procedures, the body temperature was monitored continuously using a rectal probe and was maintained at 37.0°C±0.5°C using a heating pad and lamp. The mean arterial blood pressure, pH, and arterial blood gases were measured before the suture was inserted, after 30 min of MCAO, and after 15 min of reperfusion. The vessels of the sham-operated rats were exposed without occlusion of the middle cerebral artery. At the beginning of reperfusion, a blinded observer tested the rats for neurological deficits based on a previously described scoring system as follows: 0, no neurological deficits; 1, failure to fully extend the right forepaw; 2, circling to the right; 3, falling to the right; and 4, unable to walk spontaneously and exhibiting depressed levels of consciousness. In these experiments, we used only rats presenting with scores of 2 or 3.

### Mitochondrial DNA Quantification

**Figure 1:**
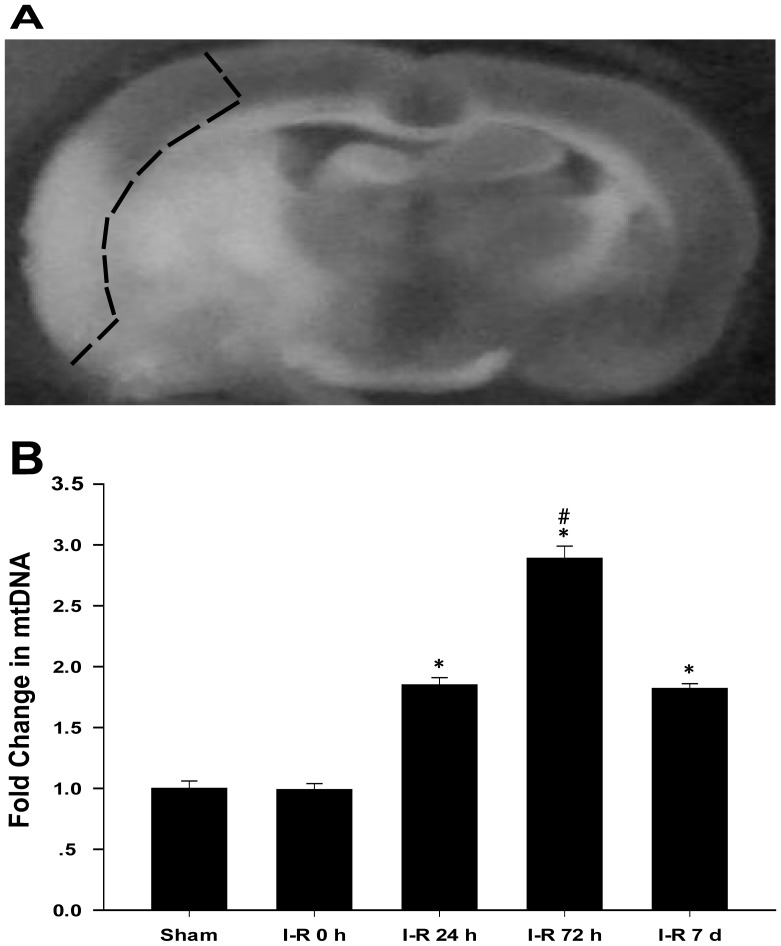
Change in the relative amounts of mtDNA after I-R. (A) Cortical tissue samples indicated by dotted line were used for PCR, Western Blot examination, citrate synthase activity assay, and morphology observation. (B) The relative amounts of cortical mtDNA were measured using quantitative real-time PCR in the sham group and the I-R 0 h, I-R 24 h, I-R 72 h, and I-R 7 d groups. The data are expressed as the means±SD. *P<0.05 versus the I-R 0 h and sham groups; #P<0.05 versus the I-R 24 h and I-R 7 d groups.

The mitochondrial DNA copy number was measured using real-time PCR as described in a previous study [23]. The animals anesthetized using 4% halothane and were decapitated at 0 h, 24 h, 72 h, and 7 d after reperfusion following 2 h of MCAO or sham operation, and the cortical lesioned tissue was identified as previously described [24]. The lesioned tissue, which included the cortical focal and perifocal tissues, was separated rapidly from the unaffected brain regions (Fig. 1A). Total DNA was extracted from the brain tissue using a QIAamp DNA extraction kit (Qiagen, Shanghai, China). In total, 10 ng of genomic DNA was used for amplifying mtDNA and nuclear DNA markers. The mtDNA was amplified using primers specific for the mitochondrial cytochrome b (Cyt B) gene, and the mitochondrial DNA copy number was normalized to the nuclear DNA copy number by the amplifying the β-actin nuclear gene. The oligonucleotides (probes) for TaqMan PCR were labeled with the fluorescent reporter dye FAM (6-carboxyfluorescein) at the 5′ end and with fluorescent BHQ-1 dye at the 3′ end. Real-time PCR amplification was performed using a LightCycler™ 480 II Probes Master kit (Roche Applied Science, Mannheim, Germany). The fluorescence threshold (Ct) value was calculated using LightCycler™ 480 II Probes system software (Roche Applied Science, Germany), and the 2^−ΔΔCt^ method was used to calculate the relative levels of expression [25]. The primer and probe sequences are listed in Table 1.

**Table 1:** mitochondrial DNA Sequences.

Gene	Primer sequence (5′ to 3′)	
CytB	Forward primer:	TCCTGCATACTTCAAAACAACG
	Reverse primer:	AACATTCCGCCCAATCACCCAAA
	Probe:	AACATTCCGCCCAATCACCCAAA
β-actin	Forward primer:	CTATGTTGCCCTAGACTTCGAGC
	Reverse primer:	TTGCCGATAGTGATGACCTGAC
	Probe:	CACTGCCGCATCCTCTTCCTCCC

### Electron Microscopy

Brains were removed at 0 h, 24 h, 72 h, and 7 d after I-R insult or sham operation, and a section of the cortex was cut into pieces measuring approximately 1 mm^3^ and processed as previously described [26]. The small blocks were placed in an ice-cold fixative (2% glutaraldehyde in 0.1 M sodium cacodylate buffer, pH 7.4) for 5 h. The samples were subsequently washed extensively with 0.1 M cacodylate buffer, post-fixed for 2 h with 2% OsO_4_/0.1 M cacodylate buffer, dehydrated in ethanol, block-stained with uranylacetate, and embedded in Epon. Ultrathin sections were collected on copper grids, double stained with uranylacetate and lead citrate, and examined using a Tecnai G2 Transmission Electron Microscopy (FEI, Portland, OR, USA). All analyses were performed in a blinded and non-biased manner. To measure the mitochondrial number, 15 randomly selected areas per animal, which included large neuronal-like nuclei covering approximately one-quarter of the visible image, were imaged at 8200× magnification and counted using minor modifications to a previously described method [21].

### Semi-quantitative RT-PCR

Rats from the sham group and each I-R group were anesthetized using 4% halothane and subsequently decapitated. The cortical lesioned tissue was immediately isolated from the brain tissue on an ice-cold glass stage. Total RNA was extracted using the RNA isolation reagent TRIzol (Invitrogen Life Technologies, Beijing, China), and reverse transcription was performed using oligo (dT) primers with a ThermoScript RT-PCR System (Invitrogen Life Technologies, Beijing, China) according to the manufacturer’s instructions. The primer pairs for rat peroxisome proliferator-activated receptor coactivator-1α (PGC-1α), nuclear respiratory factor-1 (NRF-1), mitochondrial transcription factor A (TFAM), and β-actin were derived from EMBL/GenBank sequences using computer analysis software. The forward and reverse primer sequences are provided in Table 2. The PCR reactions comprised 35 cycles for PGC-1α, NRF-1, and TFAM and 30 cycles for β-actin. The PCR cycling conditions for these genes were as follows: 30 s at 94°C for denaturing, 30 s at 56°C for annealing, and 30 s at 72°C for extension. In addition, 30 s at 94°C for denaturing was added before the cycling period, and 7 min at 72°C was added at the end of the cycling period for extension. The amplified DNA fragments were 121 bp for PGC-1α mRNA, 92 bp for NRF-1 mRNA, 329 bp for TFAM mRNA, and 207 bp for β-actin mRNA. The PCR products were run on a 2% agarose gels and visualized under UV light. The band densities were determined using ImageJ software.

**Table 2:** RT-PCR primer sequences.

Gene	Forward primer (5′ to 3′)	Reverse primer (5′ to 3′)
PGC-1α	GTGCAGCCAAGACTCTGTATGG	GTCCAGGTCATTCACATCAAGTTC
NRF-1	TTACTCTGCTGTGGCTGATGG	CCTCTGATGCTTGCGTCGTCT
TFAM	GCCTGTCAGCCTTATCTGTATTCC	CTTTCTTCTTTAGGCGTTTCTGC
β-actin	CACCCGCGAGTACAACCTT	CCCATACCCACCATCACACC

### Western Blotting

Cortical tissues were harvested at 0 h, 24 h, 72 h, and 7 d after I-R insult or sham operation. Whole-cell protein (WC) and the mitochondrial fraction (FM) were extracteds and Western blot analyses were performed as previously described [27], [28], [29]. Briefly, for detection of whole-cell protein, after homogenization, the cortical tissues were lysed in 300 μl RIPA buffer (50 mmol/L Tris-HCl [pH 7.4], 150 mmol/L NaCl, 1 mmol/L PMSF, 1 mmol/L EDTA, 1% Triton X-100, 0.5% sodium deoxycholate, and 0.1% SDS). The lysates were cleared by centrifugation at 13,000×*g* for 15 min. Protein concentrations of the samples were normalized in supernatant using a BCA protein assay kit (catalog no. 23227; Pierce, Rockford, IL, USA). To detect the mitochondrial fraction, after homogenization, the cortical tissues were lysed in five volumes of buffer (20 mM HEPES, 1.5 mM MgCl_2_, 10 mM KCl, 1 mM EDTA, 1 mM EGTA, 250 mM sucrose, 0.1 mM phenylmethylsulfonylfluoride, 1 mM dithiothreitol, and proteinase inhibitor cocktail tablets; pH 7.9). The samples were further centrifuged at 750×*g* for 15 min at 4°C to separate the sample into supernatant A and pellet A. Supernatant A, which contained the cytosolic/mitochondrial protein fraction, was further centrifuged at 16,000×*g* for 30 min at 4°C to separate supernatant B from pellet B. Pellet B was used as the mitochondrial fraction after resuspension in the buffer. The protein concentrations of the samples were normalized using a Bradford protein assay kit (Beyotime, Shanghai, China). Both the WC protein and the mitochondrial protein were denatured in SDS gel-loading buffer (100 mM Tris-HCl, 200 mM dithiothreitol, 4% SDS, 0.2% bromophenol blue, and 20% glycerol) at 95°C for 5 min and separated on 12% SDS polyacrylamide gels (20 μg per sample). After electrophoresis, the protein obtained from each sample was transferred to polyvinylidene difluoride membranes (catalog no. RPN2020F; GE Healthcare, Beijing, China). The blots were rinsed with 1X Tris-buffered saline (TBS) and 0.1% Tween (TBST) and were blocked with 5% skim milk for 1 h. The membranes were probed overnight at 4°C with a primary antibody against cytochrome C oxidase subunit IV (COX-IV, 17 kDa) (1∶1,000; catalog no. 4850; Cell Signaling Technology Inc., Massachusetts, MA, USA) and cytochrome C oxidase subunit I (COX-I, 40 kDa) (1∶1000; catalog no. ab14705; Abcam Inc., Cambridge, UK). As a loading control, the blots were probed overnight at 4°C with a primary antibody against β-actin (43 kDa) (1∶1,000; catalog no. SC-47778; Santa Cruz Biotechnology Inc., CA, USA). The specificity of each antibody was confirmed using preabsorption experiments. After overnight staining, the blots were washed three times in TBST for 10 min. The membranes were incubated for 1 h with horseradish peroxidase-conjugated secondary antibody for COX-I (1∶5,000; catalog no. SC-2005; Santa Cruz Biotechnology, Inc., CA, USA), COX-IV (1∶5,000; catalog no. SC-2004; Santa Cruz Biotechnology Inc., CA, USA), and β-actin (1∶5,000; catalog no. SC-2004; Santa Cruz Biotechnology Inc., CA, USA). Subsequently, the protein bands were visualized with ECL (catalog no. RPN 2109; GE Healthcare, Beijing, China), and the immunoreactivity was quantified using densitometry.

### Citrate Synthase Activity

Cortical tissues were harvested at 0 h, 24 h, 72 h, and 7 d after I-R insult or sham operation. Citrate synthase activity was measured using the Citrate Synthase Kit (catalog no. GMS50130; GENMED, Shanghai, China). Arbitrary activity units were calculated according to the manufacturer’s instructions.

### Histological Analysis

Cerebral damage was assessed via the histological examination of brain sections of the cerebral cortex from the sham group and from each I-R group. The animals were deeply anesthetized using chloral hydrate and transcardially perfused with 200 mL of heparinized 0.9% saline followed by 500 mL of 4% paraformaldehyde in 0.1 mol/L phosphate-buffered saline (pH 7.4). The rats were then decapitated, and the brains were dissected out and immersed in 4% paraformaldehyde for 3 d. The brain were then processed for paraffin embedding, and sectioned (5 μm thick) on a rotary microtome. Coronal sections of the cortex were selected and processed for hematoxylin and eosin (HE) staining.

### Statistical Analysis

Quantitative data were expressed as the means±standard deviation. Statistical analyses were performed using Student’s *t* test or a one-way ANOVA followed by a Fisher protected least significant difference (PLSD) post-hoc test. Values of P<0.05 were considered statistically significant.

## Results

### Physiological Parameters

Physiological parameters including the mean arterial blood pressure, pH, core temperature, and arterial blood gas tension were monitored and maintained within normal ranges for each of the experimental animals (Table 3). At the beginning of the reperfusion period, the rats were tested for neurological deficits by a blinded observer who used the following previously described scoring system: 0, no neurological deficits; 1, failure to fully extend the right forepaw; 2, circling to the right; 3, falling to the right; and 4, unable to walk spontaneously and exhibiting depressed levels of consciousness. No neurological deficits were observed in the sham-operated rats; however, significant deficits scored as 2 or 3 on the rating scale were displayed by the rats subjected to MCAO.

**Table 3:** Physiological parameters.

Parameter	Time	Group				
		Sham	I-R 0 h	I-R 24 h	I-R 72 h	I-R 7 d
MAP (mm Hg)	Baseline	98±9	98±6	99±4	99±1	99±3
	Ischemia	95±2	95±4	95±4	94±5	94±2
	Reperfusion	93±7	93±4	93±1	96±2	94±7
PaO_2_ (mm Hg)	Baseline	167±9	162±6	167±3	169±8	164±5
	Ischemia	157±10	161±7	164±4	159±8	162±5
	Reperfusion	162±4	163±3	161±4	165±4	164±4
PaCO_2_ (mm Hg)	Baseline	35±3	35±2	35±3	34±2	35±2
	Ischemia	36±1	36±1	37±1	36±2	36±1
	Reperfusion	36±2	36±1	37±2	37±1	37±1
pH	Baseline	7.38±0.02	7.38±0.02	7.39±0.02	7.37±0.02	7.38±0.01
	Ischemia	7.38±0.02	7.39±0.02	7.38±0.01	7.39±0.02	7.38±0.03
	Reperfusion	7.38±0.02	7.38±0.02	7.39±0.01	7.39±0.02	7.38±0.03
Rectal temperature	Baseline	36.7±0.2	36.9±0.1	36.8±0.2	36.7±0.2	36.7±0.2
	Ischemia	36.9±0.1	36.8±0.1	36.8±0.2	36.8±0.2	36.8±0.1
	Reperfusion	36.7±0.2	36.8±0.2	36.7±0.2	36.7±0.2	36.8±0.1

All values are presented as the mean±SD. Arterial blood gas tensions include PaO_2_, PaCO_2_, and pH.

MAP, mean arterial pressure; PaO_2_, arterial oxygen pressure; PaCO_2_, arterial carbon dioxide pressure.

### Changes in the mtDNA Content

mtDNA replication is necessary for mitochondrial biogenesis and is a typical marker used for this process. The relative abundance of mitochondria in the reperfused cerebral cortex was determined by measuring the mtDNA content using quantitative real-time PCR. No significant difference between the sham group and the I-R 0 h group was observed. An increase in the relative cortical mtDNA content at 24 h after reperfusion was observed compared with the sham group (I-R 24 h: 1.85-fold vs. sham). The mtDNA content increased to the greatest level at 72 h after reperfusion (I-R 72 h: 2.89-fold vs. sham). The mtDNA content was reduced in the I-R 7 d group, but relative values remained higher than the control level (I-R 7 d: 1.82-fold vs. sham) (Fig. 1B).

### Changes in the Number of Mitochondria

**Figure 2:**
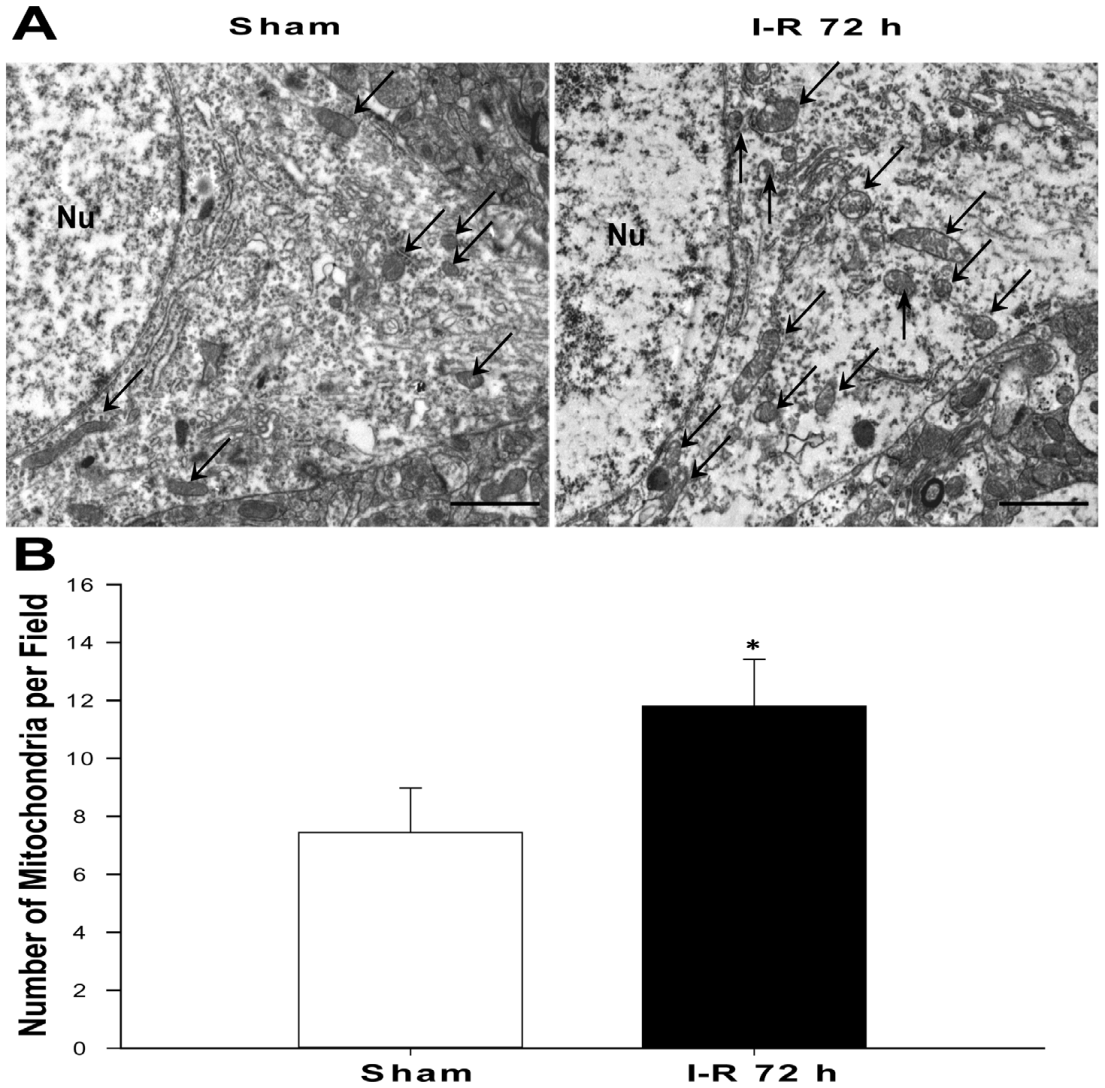
Change in the number of mitochondria after I-R. (A) Transmission electron microscopy images of a section from the cortex showing the nucleus (Nu) surrounded by relatively uniform and compact mitochondria (arrowheads). Scale bars: 2 μm. (B) A histogram quantifying the number of mitochondria in the cortical cells. Magnification of the brain sections, 8200x. *P<0.05 versus the sham group.

Mitochondrial biogenesis rates reflect the regulation of the mitochondrial number. Electron micrographs showed that the mitochondria from the sham group were distributed throughout the cytoplasm and that they had intact inner and outer membranes and well-defined cristae. The mitochondria displayed intact inner and outer membranes and well-defined cristae in the 72 h group. However, individual mitochondria showed mild swelling. The mitochondria exhibited prominent heterogeneity in size and density in the surviving cells of this group but appeared to have proliferated compared with the sham group (Fig. 2A). Quantification of the number of mitochondria confirmed this occurrence of an increase at 72 h after reperfusion following focal cerebral ischemia (Fig. 2B).

### Changes in Mitochondrial Biogenesis Factors

**Figure 3:**
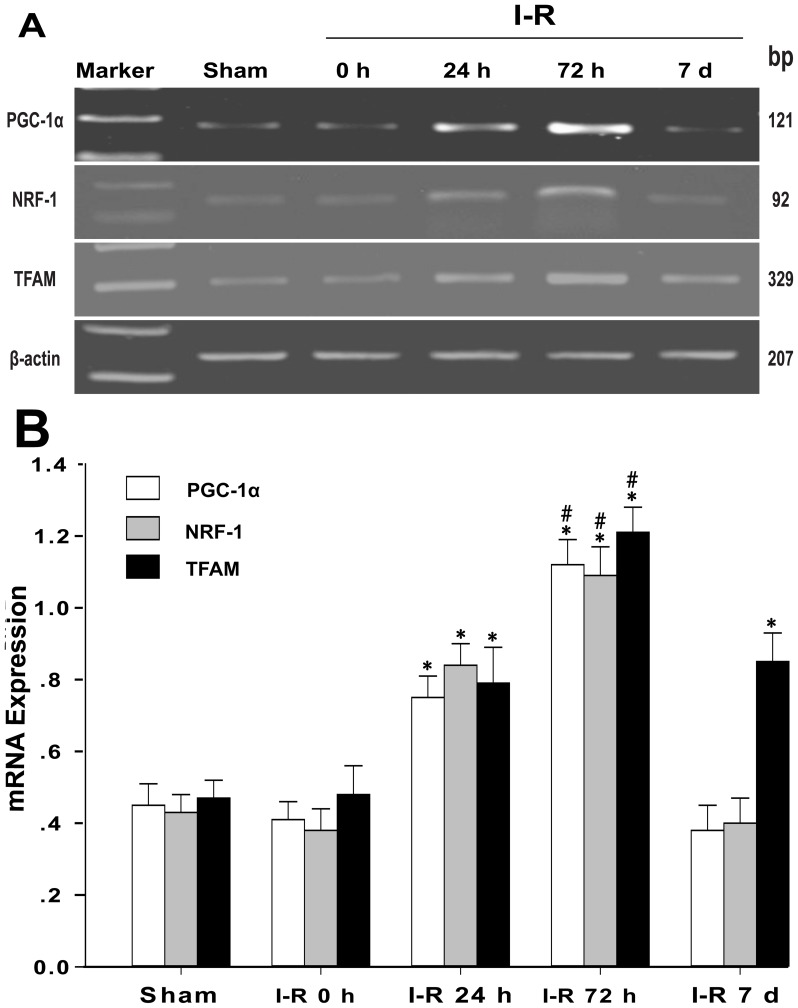
mRNA expression of the mitochondrial biogenesis factors in the cortex. (A) Representative agarose gel of PGC-1α, NRF-1, and TFAM RT-PCR products prepared from the mRNA of the sham group or the I-R 0 h, I-R 24 h, I-R 72 h, and I-R 7 d groups. (B) The histogram shows the semi-quantitative measurement of the PCR products in (A), which were obtained using densitometric analysis. The data are expressed as the means±SD. *P<0.05 versus the I-R 0 h and sham groups; #P<0.05 versus the I-R 24 h and I-R 7 d groups.

The transcription and replication of mtDNA is controlled by nucleus-encoded transcription factors and co-activators. Low levels of PGC-1α, NRF-1, and TFAM mRNA were observed in the cortex of the sham group (sham: PGC-1α = 0.44±0.06, NRF-1 = 0.43±0.05, and TFAM = 0.47±0.05). In the I-R 0 h group, PGC-1α, NRF-1, and TFAM mRNA expression was not significantly induced after 2 h of MCAO (I-R 0 h: PGC-1α = 0.40±0.05, NRF-1 = 0.38±0.06, and TFAM = 0.48±0.08). However, in contrast with the I-R 0 h group, the expression of PGC-1α, NRF-1, and TFAM mRNA increased in the cortex after 24 h of reperfusion (I-R 24 h: PGC-1α = 0.75±0.06, NRF-1 = 0.84±0.06, and TFAM = 0.79±0.09). The mRNA levels continued to increase and peaked at 72 h of reperfusion (I-R 72 h: PGC-1α = 1.12±0.07, NRF-1 = 1.09±0.08, and TFAM = 1.21±0.07). In the I-R 7 d group, the PGC-1α and NRF-1 mRNA expression returned to the control levels; however, TFAM mRNA expression remained higher compared to the I-R 0 h group (I-R 7 d: PGC-1α = 0.38±0.07, NRF-1 = 0.40±0.07, and TFAM = 0.85±0.08) (Fig. 3A and B).

### Changes in Mitochondrial Protein Expression

**Figure 4:**
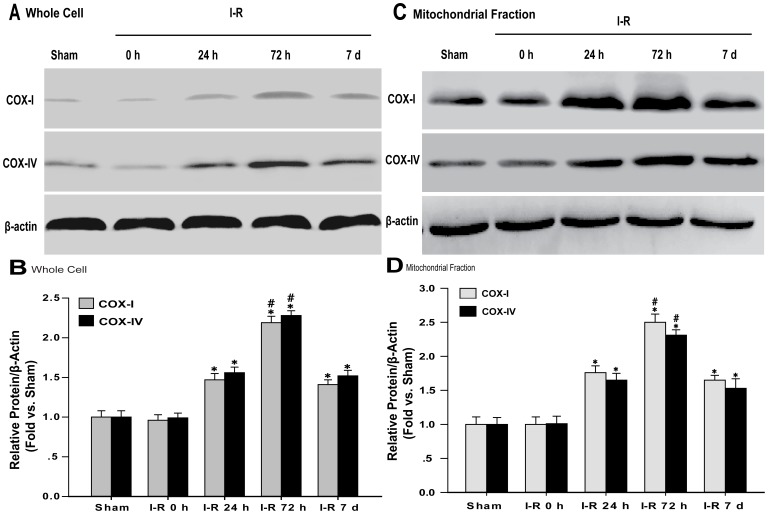
Expression of COX-I and COX-IV in the cortex. (A) Western blots showing representative COX-I and COX-IV protein expression in the whole-cell protein. β-actin was used as a control. (B) Relative analysis of the COX-I and COX-IV protein levels in the sham group and the I-R 0 h, I-R 24 h, I-R 72 h, and I-R 7 d groups. (C) Western blots showing the representative COX-I and COX-IV protein expression in the non-synaptic mitochondrial fraction. (D) Relative analysis of the COX-I and COX-IV protein levels of mitochondrial fraction in the sham group and in the I-R 0 h, I-R 24 h, I-R 72 h, and I-R 7 d groups. The data are expressed as the means±SD. *P<0.05 versus the I-R 0 h and sham groups; #P<0.05 versus the I-R 24 h and I-R 7 d groups.

The majority of mitochondrial proteins are encoded by nuclear DNA genes, and only 13 proteins are encoded by mtDNA genes. We examined the expression of the mitochondrial-specific proteins COX-I, which is encoded by mtDNA, and COX-IV, which is encoded by nuclear DNA. No significant difference in the expression levels of COX-I and COX-IV between the I-R 0 h group and the sham group was observed in WC and FM. At 24 h after reperfusion, expression levels of the COX-I and COX-IV proteins showed a significant increase in the WC and FM (I-R 24 h: COX-I: 1.47-fold [WC] 1.76-fold [FM]; COX-IV: 1.56-fold [WC], 1.65-fold [FM]). At 72 h after reperfusion, the expression of the COX-I and COX-IV proteins was greatly increased in the WC and FM (I-R 72 h: COX-I: 2.19-fold [WC], 2.50-fold [FM]; COX-IV: 2.38-fold [WC], 2.31-fold [FM]). At 7 d after reperfusion, the expression levels of the COX-I and COX-IV protein in the WC and FM moderately and gradually decreased compared with the levels observed at 72 h of reperfusion. However, the expression levels of COX-I and COX-IV protein remained higher compared with the sham group (I-R 7 d: COX-I = 1.41-fold [WC], 1.65-fold [FM]; COX-IV: 1.52-fold [WC], 1.53-fold [FM]) (Fig. 4A, B, C and D).

### Changes in the Citrate Synthase Activity

**Figure 5:**
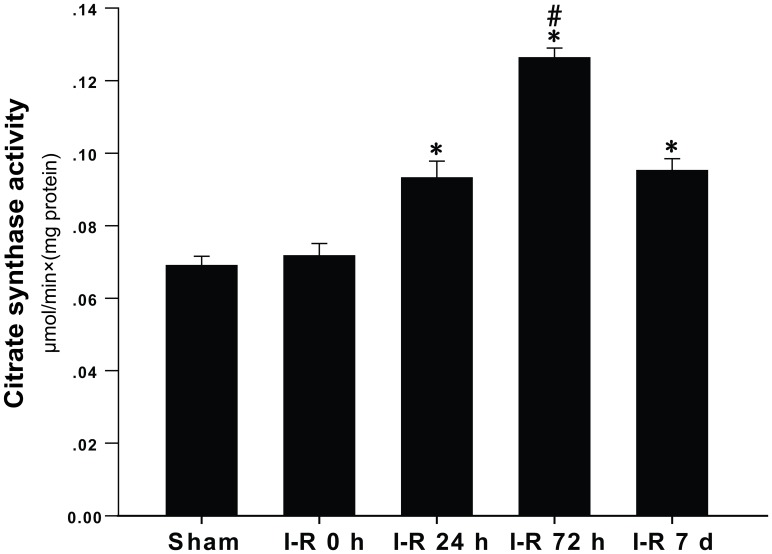
Changes in the citrate synthase activity. The citrate synthase activity was expressed as (μmols of substrate transformed)×min^−1^×(mg protein)^−1^ of citrate synthase. The data are expressed as the means±SD. *P<0.05 versus the I-R 0 h and sham groups; #P<0.05 versus the I-R 24 h and I-R 7 d groups.

Citrate synthase is a pacemaker enzyme of the citric acid cycle and is typically used as a quantitative marker for the content of intact mitochondria. No significant difference in citrate synthase activity between the I-R 0 h group and the sham group was observed (sham: 0.069±0.003 μm⋅min^−1^⋅mg protein^−1^; I-R 0 h: 0.072±0.004 μm⋅min^−1^⋅mg protein^−1^). The cortical citrate synthase activity began to increase at 24 h after I-R injury (I-R 24 h: 0.093±0.005 μm⋅min^−1^⋅mg protein^−1^), and this tendency was exacerbated at 72 h after I-R injury (I-R 72 h: 0.126±0.003 μm⋅min^−1^⋅mg protein^−1^). The citrate synthase activity decreased at 7 d after I-R injury but maintained high levels compared with the I-R 0 h group (I-R 7 d: 0.095±0.004 μm⋅min^−1^⋅mg protein^−1^) (Fig. 5).

### Histological Changes

**Figure 6:**
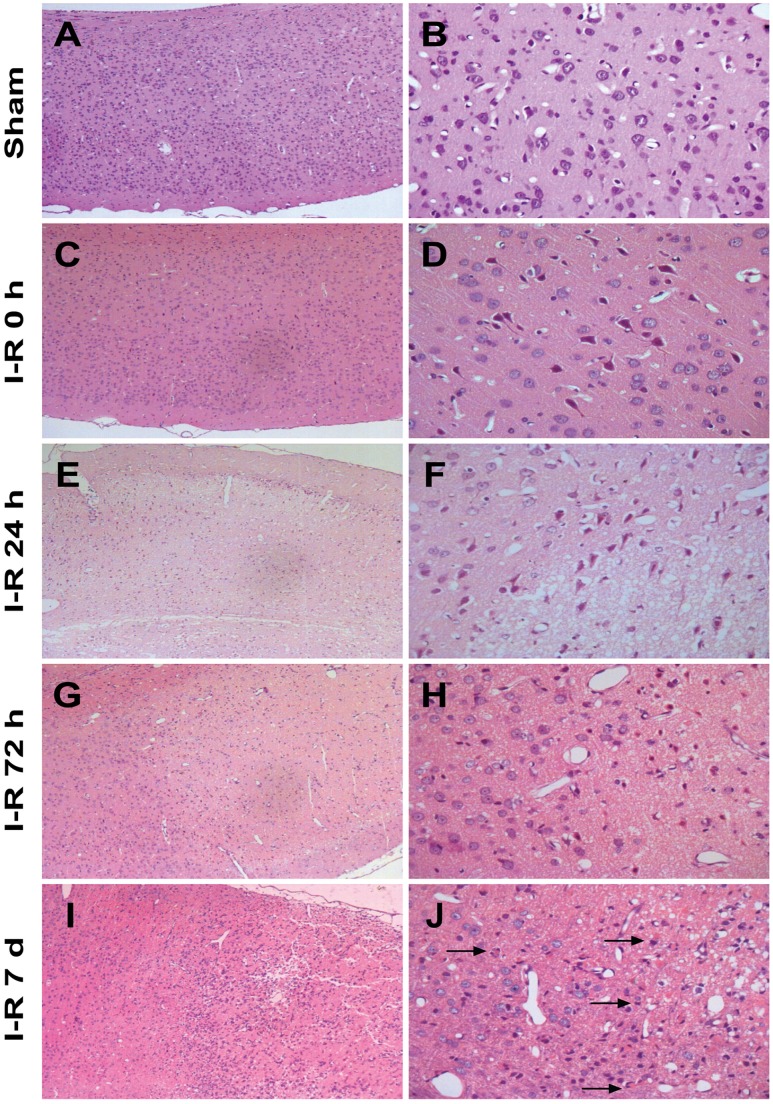
Representative histological characteristics of the cortical brain sections assessed using HE staining. (A and B) The cortical sections from the sham group revealed normal cortical tissue. (C and D) The cortical sections from the I-R 0 h group displayed cortical tissue injury. (E and F) The cortical sections from the I-R 24 h group demonstrated aggravated cortical tissue injury. (G and H) The cortical sections from the I-R 72 h group showed improved residual neurons in the damaged cortex. (I and J) The cortical sections from the I-R 7 d group showed an increased number of cells similar to glial cells in that they appeared round or elongated (arrowheads).

No morphological alterations were observed in the cortical neurons of the sham group (Fig. 6A and B). After 2 h of MCAO, a small number of neurons appeared damaged, as indicated by cell shrinkage, nuclear condensation, and fragmentation (Fig. 6C and D). Compared to the I-R 0 h group, a greater number of damaged neurons were evident in the group that experienced at 24 h reperfusion after 2 h of MCAO. In this group, the extent of damage was increased, and cytoplasmic eosinophilia was observed (Fig. 6E and F). In contrast with the I-R 24 h group, the structure of the residual neurons was improved in the I-R 72 h group, as demonstrated by the present membranes and nuclei. However, the shrunken angular neurons present at 24 h became ghost cells at 72 h (Fig. 6G and H). In the 7 d group, more neurons displayed normal cell-structure integrity. An increased number of the cells that resembled glial cells and that appeared round or elongated were observed. Increased astrocytic glial fibrillary acidic protein reactivity was observed at the interface between the focal and the perifocal brain tissue in the I-R 7 d group (Fig. 6I and J).

## Discussion

In this study, we primarily observed that the characteristic changes of increased mitochondrial biogenesis occurred continuously throughout the reperfusion period following 2 h of focal cerebral ischemia in rats. Reperfusion following focal cerebral ischemia led to a significantly increased cortical mtDNA content and an increased number of mitochondria. The increased mitochondrial biogenesis was also indicated by the higher expression of three genes critical for the transcriptional regulation of mitochondrial biogenesis – PGC-1α, NRF-1, and TFAM – and the increased levels of mitochondrial proteins COX-I and COX-IV. These results indicate that mitochondrial biogenesis increased rapidly after reperfusion following focal cerebral ischemia and suggest that mitochondrial biogenesis is a novel component of the repair mechanisms that occurs following reperfusion injury.

Mitochondria are the workhorses of cell metabolism. The number, structure, and function of mitochondria differ between cell types and tissues based on their energy demand [30], [31]. Neurons have a high requirement for energy; therefore, mitochondrial homeostasis is necessary to maintain neuronal function. Disruptions of mitochondrial function are associated with neurological diseases [32], [33], [34]. Several studies have shown that ischemia disrupts mitochondrial function and that the respiratory capacity is reduced in MCAO-damaged mitochondria in rats [24]. Oxidative stress is increased in the ischemic and post-ischemic brain [35]. High levels of matrix calcium induce changes in mitochondrial permeability [36]. However, mitochondrial biogenesis is also induced by ischemic or hypoxic damage, and this mitochondrial biogenesis may increase the abundance of mitochondria and maintain their aerobic set-point in the face of declining function [5]. A previous study showed that enhancing mitochondrial biogenesis reduces ischemic cerebral injury [37]. Thus, this evidence suggests that increased mitochondrial biogenesis is a protective signal induced by ischemic injury. Although many mitochondria were damaged after reperfusion following 2 h of MCAO, we also, surprisingly, observed a large number of undamaged mitochondria. In the present study, we demonstrated a clear time course of mitochondrial biogenesis during reperfusion. The number of mitochondria increased significantly in neurons at 72 h after reperfusion. The structure of the mitochondria was normal in that they displayed intact inner and outer membranes and well-defined cristae. The rate of mitochondrial biogenesis was increased in the surviving cells, including neurons and glia [38].

Endosymbiotic mitochondria possess their own genome, and the replication of mtDNA denotes mitochondrial biogenesis [39]. Our study revealed significantly increased mtDNA copy numbers at 24 h, 72 h, and 7 d after reperfusion following 2 h of MCAO. The results obtained in the present study indicate that post-ischemic reperfusion induced the increase in mitochondrial biogenesis. A previous study reported that the reduced mtDNA content is restored after reperfusion following 30 min focal cerebral ischemia [40], and our findings appear to be consistent with those results. However, many differences exist between that previous study and our study. The previous study used transcranial occlusion of the middle cerebral artery, and both common carotid arteries were clamped with vascular clips. Our study used filamental occlusion of the middle cerebral artery. The methods utilized in the previous study resulted in a greater number of irreversibly damaged cells during the ischemic period. Furthermore, the mtDNA content was not restored to near non-ischemic levels after 90 min of MCAO, but a restore in mtDNA content was observed 24 h after I-R injury. In our study, we observed an increase in mtDNA at this time point. However, the mtDNA content increased greatly in 72 h after I-R injury and decreased moderately at 7 d after I-R injury.

Mitochondrial biogenesis requires the coordinated expression of nuclear and mitochondrial genes [41]. The transcription and replication of mtDNA is controlled by nuclear-encoded transcription factors and co-activators, but nuclear gene expression is influenced by mitochondrial signals [42], [43], [44]. PGC-1α was first identified as the major regulator of mitochondrial biogenesis [45]. PGC-1α is a master regulator of ROS scavenging enzymes, including manganese superoxide dismutase 2 and uncoupling protein 2, and both mitochondrial proteins may contribute to neuronal survival [8], [16]. Oxidative stress has been strongly implicated as an important factor in the development of I-R injury, and the expression of PGC-1α is increased by abundant ROS. The increased intracellular calcium concentration up-regulates the expression of PGC-1α, causing an increased mitochondrial content [46]. A previous study showed that activation of the CaMKIV/PGC-1α signaling pathway promotes mitochondrial biogenesis in the hippocampal CA1 subfield [47]. Therefore, excessive ROS and calcium overload are induced by reperfusion injury [48], [49], which may up-regulate the expression of PGC-1α. In the present study, we observed that the expression of PGC-1α mRNA increased following ischemia-reperfusion injury. PGC-1α is also a co-transcriptional regulation factor that induces mitochondrial biogenesis by activating a group of transcription factors, including nuclear respiratory factor 1 (NRF1) and nuclear respiratory factor 2 (NRF2), which activate mitochondrial transcription factor A (TFAM). A previous study reported that ischemia-induced NRF1 increases at 72 h after I-R injury [50]. In the present study, we also observed that NRF-1 expression increased after reperfusion following 2 h of MCAO. NRF-1 expression increased greatly at 72 h after reperfusion, however, the level of NRF-1 only increased moderately at 24 h and 7 d following ischemia-reperfusion. In addition, the results obtained in the current study revealed that the expression of TFAM in nerve cells was modified in a time-dependent manner during reperfusion. Both the mtDNA copy number and TFAM expression levels increased after 24 h of reperfusion, peaked after 72 h, and remained increased after 7 d of reperfusion. These results also indicate that the amount of mtDNA is directly proportional to the total TFAM levels [51]. At 7 d after reperfusion, the TFAM and the mtDNA copy number decreased but remained greater than the control levels. By contrast, the expression of NRF-1 returned to baseline levels before reperfusion. This result reflects the fact that the transcriptional regulation of mitochondrial biogenesis occurs upstream to a change in the mtDNA copy number. All these results indicated that post-ischemic reperfusion induces an increased expression of nuclear genes associated with mitochondrial biogenesis.

Over the course of evolution, the majority of mitochondrial genes have beentransferred to and integrated into the genome of the host cell [52]. Therefore, most mitochondrial proteins are encoded by nuclear DNA and are imported into the organelle. The mtDNA encodes 13 proteins that form part of the multi-subunit complexes of the oxidative phosphorylation system. COX-I is encoded by nuclear DNA, and COX-IV is encoded by mtDNA. In the current study, we simultaneously examined the levels of COX-I and COX-IV in the WC and FC. The levels of COX-I and COX-IV increased after 24 h of reperfusion in the FM. The expression of both proteins peaked at 72 h of reperfusion following 2 h of MCAO in the FM. After 7 d of reperfusion, the levels of COX-IV and COX-I decreased in the FM but remained higher than the control levels. This change in protein expression suggests that mitochondrial biogenesis was stimulated by reperfusion in the non-synaptic mitochondrial fraction. Meanwhile, in the whole-cell protein, the changes in COX-IV and COX-I levels were in accord with the changes in the non-synaptic mitochondrial fraction. The changes in the COX-IV and COX-I levels were also consistent with the changes in the mtDNA copy number. Both the copy number and the protein expression levels were decreased at 7 d after ischemic-reperfusion but remained greater than the control levels. Manoli et al. proposed that prolonged challenges to mitochondrial homeostasis exceed the mitochondrial reserves, which decreases mitochondrial biogenesis at 7 d of reperfusion in irreversibly damaged cells [53]. By contrast, the mitochondrial abundance might have been reduced to decrease the production of ROS at 7 d in the reversibly damaged cells. The changes in the mitochondrial abundance, mtDNA copy number, and expression of respiratory genes have been associated with altered intracellular levels of ROS [54].

To determine whether there was a concomitant increase in mitochondrial function, citrate synthase activity was measured after I-R injury. Citrate synthase activity is considered a biochemical marker of mitochondrial mass [55]. In our current study, we observed that the citrate synthase activity was not significantly changed by 2 h of ischemia but was increased after reperfusion following 2 h of MCAO. The changes in the citrate synthase activity were consistent with the changes we observed in the number of mitochondria and in the amount of mtDNA. The mitochondrial function improved with the increased mitochondrial biogenesis. In previous studies, citrate synthase activity was not significantly changed by ischemia [21], [56], and these findings are consistent with our results. However, another study found that citrate synthase activity is increased by ischemia in old rats [57]. This difference is mainly due to the age difference of the animals used in the experiments because Villa et al. have already established that aging affects the catalytic properties of mitochondrial enzymes [58].

Mitochondrial biogenesis may be an endogenous protective signal. Acute stress results in increased mitochondrial biogenesis to meet the increased energy demands of the cell [53], [54]. In addition, studies have indicated that ischemic injury may be reduced by the increasing in mitochondrial biogenesis. One study reported that exercise after ischemic injury promotes mitochondrial biogenesis, reduces the brain ischemic area, and improves neurological symptoms [59]. Another study showed that acetyl-L-carnitine induces neuroprotection through increasing mitochondrial biogenesis [60]. These recent studies indicate that preventing a reduction in the number of mitochondria may be beneficial for reducing the infarct size caused by MCAO; in the present study, this idea was demonstrated by the changes in the neuronal damage detected by HE staining. The structure of the residual neurons was improved in the I-R 72 h group, as indicated by the visible membranes and nuclei. A greater proportion of neurons displayed normal cell-structure integrity in the 7 d group. Overall, these results strongly suggest that the regulation of mitochondrial biogenesis promotes the recovery of neural lesions caused by I-R injury. However, we have not investigated the mechanism mediated by changes in mitochondrial biogenesis that influenced the fate of the damaged neurons during reperfusion.

In conclusion, we have provided evidence that reperfusion induces a significant increase in the expression of mitochondrial biogenesis factors, which results in mitochondrial biogenesis after ischemic brain injury. Thus, reperfusion may improve brain recovery from ischemia-induced injury by regulating mitochondrial biogenesis. In addition, these data further support the notion that the stimulation or enhancement of mitochondrial biogenesis may provide a novel neuroprotective strategy in the future.

## Supporting Information

Table S1 The number of animals (total n = 168).
(DOC)
