# Supplementary material for: Reperfusion Promotes Mitochondrial Biogenesis following Focal Cerebral Ischemia in Rats
Source: PLoS One. 2014 Mar 25;9(3):e92443. doi: 10.1371/journal.pone.0092443 (PMC3965405; doi:10.1371/journal.pone.0092443)
Supplement: Table S1 — The number of animals (total n = 168). (DOC) [file pone.0092443.s001.doc]

| Table S1. The number of animals (total n=168) | | | | | |
| --- | --- | --- | --- | --- | --- |
| Analysis type | Group | | | | |
|  | Sham | I-R 0 h | I-R 24 h | I-R 72 h | I-R 7 d |
| DNA quantification | 6 | 6 | 6 | 6 | 6 |
| Electron microscopy | 6 | — | — | 6 | — |
| RT-PCR | 6 | 6 | 6 | 6 | 6 |
| Western blot analysis | 6 | 6 | 6 | 6 | 6 |
| HE staining | 2 | 4 | 4 | 4 | 4 |
| Citrate synthase activity | 6 | 6 | 6 | 6 | 6 |
| The samples of Electron microscopy and Western blot were obtained from the same animal. | | | | | |
